# A Machine Learning Approach for Predicting 30-Day Hospital Readmission in Patients with Diabetes

**DOI:** 10.3390/healthcare14091185

**Published:** 2026-04-28

**Authors:** Safaa Saad Salim, Abdullahi Abdu Ibrahim

**Affiliations:** Department of Electrical and Computer Engineering, Altınbaş University, Mahmutbey Dilmenler Caddesi, No: 26, Bağcılar, İstanbul 34217, Turkey; abdullahi.ibrahim@altinbas.edu.tr

**Keywords:** machine learning, hospital readmission, diabetes, XGBoost, clinical decision support, predictive modeling

## Abstract

**Background:** Hospital readmission among patients with diabetes remains a major challenge for healthcare systems, contributing to increased costs and adverse patient outcomes. Early identification of high-risk patients may support targeted interventions and improved care management. **Objectives:** This study aimed to develop and rigorously evaluate a machine learning framework for predicting 30-day hospital readmission in patients with diabetes using a large multi-institutional clinical dataset. **Methods:** The study utilized the Diabetes 130-US Hospitals dataset from the UCI Machine Learning Repository, comprising 101,766 hospital encounters. Data preprocessing included missing-value handling and feature engineering. Several machine learning models were evaluated, including Logistic Regression, Random Forest, XGBoost, and LightGBM, alongside a stacking ensemble model. Model performance was assessed using nested cross-validation (5 outer folds, 3 inner folds), probability calibration via Platt scaling, and statistical robustness through 1000 bootstrap resamples. Clinical utility was evaluated using decision curve analysis and clinical impact curves, while SHAP analysis was applied for model interpretability. **Results:** The stacking ensemble model achieved a nested cross-validated ROC–AUC of 0.664 and a calibrated AUC of 0.688, with a Brier score of 0.094. Risk stratification demonstrated a clear gradient between low- and high-risk groups, and decision curve analysis indicated positive clinical net benefit across relevant decision thresholds. **Conclusions:** The proposed machine learning framework provides a robust and clinically interpretable approach for predicting 30-day hospital readmission in diabetic patients, with potential utility for supporting clinical decision-making and care management.

## 1. Introduction

### 1.1. Hospital Readmission as a Healthcare Challenge

Hospital readmission remains a persistent challenge for healthcare systems worldwide. Unplanned readmissions which occur within 30 days after discharge are used as an indicator to assess both healthcare quality and efficiency [1,2]. Hospitals face increased operational costs, patient health complications, and resource pressure because of early hospital readmissions [1]. Therefore, reducing avoidable hospital readmissions has become a major goal for healthcare providers and policymakers.

Patients with chronic diseases face a high risk of repeated hospital visits. Diabetes mellitus stands out as one of the most common yet medically complex conditions affecting people worldwide [3]. Individuals with diabetes commonly develop complications including cardiovascular disease, renal impairment, infections, and metabolic instability [4]. These complications often lead to repeated hospital admissions and increase the demand for healthcare services.

The task of preventing readmission becomes more difficult because multiple factors contribute to this complex issue. The risk of readmission depends not only on the patient’s clinical condition but also on treatment management, discharge planning, and social determinants of health [5]. As a result, healthcare systems often find it difficult to accurately identify patients who have a high risk of early hospital readmission.

### 1.2. Predictive Modeling in Healthcare

Healthcare decision-making now relies on predictive modeling, which serves as a fundamental approach to enhance its results. The predictive models use historical clinical data analysis to find patterns which lead to negative outcomes, thus enabling medical professionals to treat patients earlier and optimize their resource distribution decisions [6].

Hospitals use logistic regression and other traditional statistical methods to create models that predict hospital readmission risks. The methods enable users to understand how variables affect outcomes, but they struggle to handle the intricate and nonlinear relationships present in extensive clinical data. Healthcare data requires advanced analytical methods, which enable researchers to extract complete data value from increasingly complex and diverse healthcare information [7].

Recent machine learning methods have proven to be effective for predicting clinical outcomes. Machine learning models can learn complex relationships from structured healthcare data and identify subtle patterns that may not be apparent using conventional statistical methods. Machine learning proves most effective for predicting hospital readmission, which results from multiple interconnected elements [6,8].

### 1.3. Machine Learning for Hospital Readmission Prediction

Multiple research studies have investigated how machine learning algorithms can predict hospital readmission. Three machine learning methods, random forests, gradient boosting, and neural networks, have been tested on electronic health record data and administrative datasets to achieve better results in their predictive capabilities.

Most studies that exist at the present time concentrate on predictive accuracy at the expense of crucial elements needed to assess model performance. The evaluation process for models typically relies on discrimination metrics which include area under the receiver operating characteristic curve (AUC), yet lacks assessment of probability calibration and clinical usefulness [9]. Accurate probability estimation holds critical importance because it serves as the basis for making clinical decisions based on predictions.

The present research contains another limitation because its performance estimates exceed their actual value because validation methods used in the study are not sufficient. The process of hyperparameter tuning and performance evaluation requires separate datasets, because using the same datasets for both activities will create result inaccuracies [10]. The implementation of nested cross-validation as a robust validation method provides more reliable performance estimates and reduces the risk of optimistic bias during model evaluation [10].

Machine learning models need to be interpretable because clinicians require this feature for successful clinical implementation. According to clinicians, they will trust and use predictive tools when the system shows how it makes decisions while using established clinical factors [11].

The central question of this study is therefore not whether a novel algorithm can be developed, but whether a methodologically rigorous approach applied to a large, heterogeneous administrative dataset can yield stable, well-calibrated, and clinically interpretable predictions—even under the inherent constraints of administrative data.

### 1.4. Study Objective and Contributions

To address these methodological gaps, this study makes the following commitments to rigorous evaluation:

1. A robust evaluation framework using nested cross-validation to provide unbiased performance estimation.
2. Probability calibration of model outputs using Platt scaling to improve the reliability of predicted readmission risk.
3. Clinical utility assessment through decision curve analysis (DCA) and clinical impact curves (CICs) to evaluate the real-world usefulness of the model.
4. Model interpretability using SHAP analysis combined with stability analysis to identify consistent predictors of hospital readmission.
5. Comparative evaluation of multiple machine learning models, including Logistic Regression, Random Forest, XGBoost, and LightGBM, to identify the most appropriate base classifier for calibration and interpretability analysis.

## 2. Related Work

Recent studies have explored machine learning techniques for predicting hospital readmissions among patients with diabetes. Mishra et al. created predictive models using Logistic Regression, Decision Tree, Random Forest, and XGBoost to predict 30-day readmission risk for diabetic patients. Their study utilized a clinical dataset of 352 patient records collected from a diabetes specialty clinic. The results demonstrated that XGBoost achieved the highest predictive performance, with precision of 0.84, recall of 0.87, and F1-score of 0.85, while Random Forest achieved the highest AUC-ROC value of 0.94. The research demonstrates how ensemble learning models effectively identify high-risk patients who require targeted interventions to decrease hospital readmissions [12].

Another study applied machine learning techniques to predict 30-day hospital readmission risk in pediatric patients. Silva et al. developed predictive models using several algorithms including Logistic Regression, Decision Tree, Random Forest, Gradient Boosting Machine, and XGBoost, which used demographic data, clinical information, and biochemical data from 9080 hospitalized patients. The dataset was divided into training and testing sets, and cross-validation was repeatedly used for model evaluation. The results showed that XGBoost achieved the highest predictive accuracy with an AUC score of 0.814, an 81% sensitivity rate, and a 66% specificity rate. The study discovered multiple key factors that predicted readmission risk, such as cancer diagnosis, age, red blood cell levels, leukocyte count, sodium levels, and multimorbidity. The findings demonstrate how machine learning models can assist hospitals in detecting patients at high risk of readmission [13].

The research study analyzed machine learning methods for predicting hospital readmissions within 30 days based on medical records from Massachusetts hospitals. Awe et al. created predictive models through their application of Ridge Regression and Random Forest and Gradient Boosting algorithms to structured healthcare data which they used to determine hospital readmission probabilities. The dataset contained statewide hospital data including risk-adjusted readmission rates, number of cases, and number of deaths or readmissions. The results showed that ensemble learning models achieved better results than traditional regression models because Gradient Boosting produced the highest prediction accuracy with an R^2^ value of about 0.81 and minimal prediction error. Advanced ensemble machine learning techniques successfully enhanced hospital readmission prediction accuracy which assisted healthcare decision-making processes according to the research results [14].

Researchers used machine learning to forecast 30-day hospital readmissions for diabetic patients by analyzing their electronic health records. Emi-Johnson et al. created predictive models using Logistic Regression, Random Forest, XGBoost, and Deep Neural Networks, which were constructed using the UCI Diabetes 130-US Hospitals dataset that contains 101,766 inpatient records. The dataset contained demographic data together with information about comorbidities, medication records, laboratory test results, and details about previous hospital admissions. The results showed that XGBoost achieved the best predictive performance with an AUC-ROC of 0.667, followed by Logistic Regression (0.642) and Random Forest (0.630), while Deep Neural Network achieved the lowest performance. The study used SHAP analysis to determine which predictors most affected readmission risk, including prior inpatient visits, total medications, and hospital stay duration. These findings show that ensemble machine learning models can enhance hospital readmission prediction accuracy while assisting clinical decision-making processes [15].

Another study used electronic health record data to compare different machine learning and deep learning models that predict 30-day hospital readmission rates for patients with diabetes. Liu et al. created predictive models using multiple algorithms, including Random Forest, XGBoost, Decision Tree, Support Vector Machine, Logistic Regression, KNN, Naïve Bayes, AdaBoost, Multilayer Perceptron, and LSTM. The research study utilized the UCI “Diabetes 130-US Hospitals for Years 1999–2008” dataset, which contains 101,766 inpatient encounters from 130 hospitals across the United States. The researchers applied preprocessing techniques including handling missing values, feature engineering, and feature selection using the Grey Wolf Optimizer (GWO) method, while SMOTE was used to handle class imbalance during cross-validation. The Random Forest model achieved the highest overall performance with an F1 score of 0.83 and an accuracy of 0.88, while XGBoost produced similar results. These results demonstrate the ability of ensemble machine learning models to predict hospital readmission rates among patients with diabetes [16].

Researchers studied deep learning methods that predict 30-day hospital readmission rates for Medicare patients. Li et al. developed a predictive model using Long Short-Term Memory (LSTM) networks to analyze electronic health record data. The research utilized data extracted from the MIMIC-III database, which contains clinical records from multiple hospital admissions. The model used admission information together with patient medical history and demographic data to analyze how patient health records change over time. The results demonstrated that the LSTM model achieved an average AUC score of 0.700, exceeding the baseline logistic regression model based on the LACE index, which achieved an AUC score of 0.608. The study identified important predictors influencing readmission risk, including the Charlson Comorbidity Index, length of hospital stay, and previous hospital admissions. These findings show that deep learning models can enhance hospital readmission prediction by capturing temporal patterns in clinical data [17].

Another research project examined how machine learning techniques can forecast diabetes patient outcomes who may require hospital readmission within 30 days. Shang et al. created predictive models using multiple machine learning techniques such as Random Forest, Naïve Bayes, and Tree Ensemble to analyze electronic health record data. The study used data from the Health Facts database, which includes more than 100,000 records of diabetic patients collected from 130 hospitals between 1999 and 2008. After preprocessing and normalizing the data, the researchers selected 23 critical attributes, including age, sex, admission type, number of inpatient visits, length of hospital stay, and medication usage, as risk factors for model training. The dataset was divided into training and testing sets using an 80–20% split. The results demonstrated that the Random Forest model achieved the best performance among the tested models, with an AUC score of approximately 0.661 for predicting 30-day hospital readmission. The study also identified key predictors influencing readmission risk, including admission frequency, age, diagnosis information, and emergency visits. These findings demonstrate that machine learning models can help healthcare professionals identify patients who are at high risk of hospital readmission [18].

The research work examined machine learning techniques for predicting hospital readmission among diabetic patients, but several limitations remain. Existing methods often use basic train–test evaluation approaches, which may lead to unreliable performance estimates and limit the robustness of model evaluation. Most research studies concentrate on predictive performance metrics such as accuracy and AUC, while often overlooking model calibration and clinical usefulness. In addition, the evaluation of important predictive features is often limited, which may reduce the reliability of the models in real healthcare settings.

The current study proposes a comprehensive machine learning approach for predicting 30-day hospital readmission among patients with diabetes to address these limitations. The proposed approach employs nested cross-validation for robust model evaluation, calibration techniques to improve probability estimation, and decision curve analysis to assess the clinical applicability of the developed models. The study also applies SHAP-based interpretability and feature stability analysis to identify the most reliable predictors influencing readmission risk. This integrated framework aims to provide more reliable predictions with improved clinical relevance compared to previous approaches.

## 3. Materials and Methods

### 3.1. Dataset Description and Preprocessing

#### 3.1.1. Data Source and Study Population

The research employed the “Diabetes 130-US Hospitals for Years 1999–2008” dataset, which the UCI Machine Learning Repository (University of California, Irvine, CA, USA) provided. The dataset includes 101,766 hospital visits, which researchers collected from 130 US hospitals and integrated delivery networks throughout a decade between 1999 and 2008.

The data set contains information about hospitalized patients who received treatment for diabetes mellitus through inpatient medical care that included laboratory tests and medication treatment and hospital admission that lasted less than 14 days. The dataset contains demographic information together with data on hospital admission and laboratory results and diagnostic codes and information about medications.

It should be noted that the definition of ‘patients with diabetes’ in this study follows the original dataset inclusion criteria, which required that each encounter involved a patient who received diabetes-related treatment during hospitalization, including laboratory tests or medication management. No additional filtering based on explicit ICD diabetes codes within the diag_1–diag_3 fields was applied, as these fields represent the primary, secondary, and tertiary diagnoses recorded during the encounter, which may or may not list diabetes as the principal diagnosis. This approach is consistent with the original data collection methodology described by Strack et al. (2014), the primary publication associated with this dataset [19].

#### 3.1.2. Outcome Definition

The primary endpoint of this study was early hospital readmission within 30 days of discharge.

The original dataset categorized readmission status into three groups:

- “<30” (readmitted within 30 days),
- “>30” (readmitted after 30 days),
- “NO” (no readmission).

For modeling purposes, this variable was reformulated into a binary outcome:

- Target = 1: readmitted within 30 days,
- Target = 0: otherwise.

This definition aligns with established hospital quality metrics that focus specifically on 30-day readmission risk as a performance indicator.

It should be noted that the dataset does not distinguish between planned and unplanned readmissions. The binary outcome therefore captures all readmissions occurring within 30 days, regardless of whether they were scheduled in advance or resulted from clinical deterioration. This is a recognized limitation of administrative datasets used for readmission prediction, as contemporary quality metrics typically focus on unplanned readmissions only. Future work using datasets that explicitly classify readmission type would allow a more clinically precise prediction target.

The resulting class distribution was as follows:

- Non-readmitted: 90,409 encounters (88.84%).
- Readmitted within 30 days: 11,357 encounters (11.16%).

This approximately 1:8 class imbalance reflects real-world clinical prevalence and requires appropriate modeling strategies to prevent bias toward the majority class.

#### 3.1.3. Data Cleaning and Missing Value Handling

To prevent information leakage, unique patient and encounter identifiers (encounter_id and patient_nbr) were removed prior to any modeling procedures.

As shown in Table 1, a full audit of missingness across all variables was conducted and revealed the following pattern:

Variables with near-complete missingness (weight at 96.86% and payer_code at 39.56%) were excluded from analysis. For all remaining variables, categorical missing values encoded as “?” were replaced with explicit missing values and subsequently imputed as a separate “Unknown” category. This strategy preserved structural information without introducing artificial distributional assumptions. The decision to retain missing categorical values as an explicit ‘Unknown’ category was based on three methodological considerations. First, this strategy preserves the structural information that a value was not recorded, which may itself carry predictive signal, consistent with the missing indicator method described in clinical prediction modeling literature [20]. Second, mode imputation can distort the distribution of variables with high missingness rates, particularly when data are not missing completely at random, as is commonly observed in administrative EHR datasets [20]. Third, tree-based models such as XGBoost can natively handle explicit missingness categories as informative input features, representing the least computationally intensive approach with reasonable predictive performance [21]. No numerical imputation was required, as no missing values remained in numerical features after this cleaning step.

Although payer_code exhibited lower missingness than some retained variables such as A1Cresult (83.28%), the decision to exclude it was based primarily on its administrative and financial nature rather than missingness alone. Unlike A1Cresult, which reflects glycemic control and is directly relevant to diabetes management and readmission risk, payer_code captures insurance classification and is unlikely to contribute meaningful clinical signal to the prediction model. Retaining variables with high missingness but strong clinical relevance, while excluding administratively defined variables with limited predictive value, reflects a clinically informed rather than purely statistical approach to missing data handling.

Following cleaning procedures, the dataset consisted of 101,766 encounters and 46 variables prior to feature engineering.

#### 3.1.4. Feature Engineering

To improve clinical interpretability and reduce dimensional sparsity, several domain-informed transformations were performed.

##### Age Transformation

Age was originally represented as categorical intervals (e.g., “[60–70)”). These intervals were converted into numerical midpoints to allow continuous modeling while preserving ordinal structure.

While alternative encodings were considered—including ordinal integer encoding and clinically motivated groupings such as age below 60 versus 60 years and above—the midpoint transformation was selected as it preserves the continuous ordinal structure of age bands while enabling the model to capture monotonic relationships between age and readmission risk. This approach is consistent with prior studies using this dataset and avoids the potential loss of granularity associated with binary age groupings.

To further validate this encoding choice, a comparative analysis was conducted in which age was represented using ordinal integer encoding (0–9). The midpoint encoding achieved a cross-validated AUC of 0.664, compared with 0.652 for ordinal encoding, confirming that the midpoint transformation yields superior predictive performance and that the contribution of age in the SHAP analysis reflects a stable clinical signal rather than an encoding artifact

##### Diagnostic Code Grouping

The three recorded diagnosis fields (diag_1, diag_2, and diag_3) were mapped into clinically meaningful categories based on ICD code ranges. The resulting categories included:

- Circulatory diseases;
- Respiratory diseases;
- Digestive diseases;
- Diabetes-related conditions;
- Injury;
- Cancer;
- Other.

Binary indicator variables (e.g., has_circulatory, has_diabetes) were created to capture the presence of each diagnostic group across any of the three diagnosis fields, thereby modeling comorbidity burden explicitly. The original diagnosis columns were subsequently removed to reduce multicollinearity and high-cardinality sparsity.

##### Administrative Variable Consolidation

Hospital administrative codes were grouped into clinically interpretable categories:

- Admission type: Emergency, Urgent, Elective, Other.
- Discharge group: Home, Transfer, Expired, Other.
- Admission source group: Emergency Room, Physician Referral, Transfer, Other.

These transformations reduced categorical fragmentation and improved modeling stability while preserving clinical meaning.

##### High-Cardinality Reduction

The medical_specialty variable initially contained 73 distinct categories. To reduce overfitting risk during one-hot encoding, only the ten most frequent specialties were retained, and all remaining categories were grouped under “Other,” resulting in 11 final categories.

#### 3.1.5. Final Modeling Dataset

After completion of all preprocessing and feature engineering procedures, the final dataset contained 101,766 hospital encounters and 55 predictor variables. This finalized dataset was saved as final_dataset.csv and subsequently used for model development, nested cross-validation, calibration, and clinical utility analysis.

All modeling steps were implemented within a pipeline framework to ensure reproducibility and prevent data leakage during cross-validation.

A detailed summary of all variable transformations is provided in Supplementary Table S1.

The dataset summarized in Table 2 reflects a large, real-world, multi-institutional clinical cohort with substantial heterogeneity across hospitals and patient characteristics. The study found that 30-day readmission rates reached 11.16%, which matches the hospital readmission rates that research shows for diabetes patients, thus validating the study’s population as clinically authentic.

The irregular distribution of classes, which produces a class imbalance with a ratio of approximately 1 to 8, requires researchers to use modeling techniques that handle unequal class distribution correctly. The study used a sample size of 101,766 participants which allowed researchers to establish statistical power needed for creating strong models that required nested cross-validation and calibration checking and analysis of clinical usefulness.

The dataset characteristics of this study create a foundation which researchers can use to build a prediction framework that predicts early hospital readmissions for diabetes patients in a way that remains applicable to different clinical settings.

### 3.2. Model Development and Validation Strategy

A structured machine learning approach was developed to predict 30-day hospital readmissions. All analyses were performed using Python (version 3.12.7; Python Software Foundation, Beaverton, OR, USA). The research team built a reproducible pipeline which handled all preprocessing tasks and model training processes and evaluation methods to maintain research integrity and protect against data contamination.

The modeling process consisted of four main components: (1) preprocessing and feature transformation, (2) model training with class imbalance adjustment, (3) nested cross-validation for unbiased performance estimation, and (4) post-training calibration and clinical utility evaluation.

The researchers used Extreme Gradient Boosting (XGBoost, version 3.1.2; DMLC, open source) as the main classification method because it can model nonlinear relationships and works with different types of data while proving effective with structured clinical information [22].

Although the stacking ensemble achieved a marginally higher ROC–AUC of 0.665 compared with 0.664 for XGBoost, this difference of 0.007 falls within the bootstrap confidence interval margin and is not statistically meaningful. XGBoost was therefore selected as the primary model based on its compatibility with SHAP-based interpretability and probability calibration, rather than on discriminative performance alone.

Additional models were included for comparative evaluation to empirically justify this selection. Logistic Regression was included as an interpretable linear baseline. Random Forest was selected as a well-established non-linear ensemble method with strong generalization properties. LightGBM (version 4.6.0; Microsoft Corporation, Redmond, WA, USA) was included due to its computational efficiency with large, structured datasets. A stacking ensemble was constructed to assess whether combining multiple classifiers yields meaningful performance gains over individual models.

The stacking ensemble was configured using three base learners: Logistic Regression, XGBoost, and LightGBM. A Logistic Regression model was used as the meta-learner to combine the predicted probabilities of the base classifiers. The stacking architecture was implemented using scikit-learn’s StackingClassifier (scikit-learn, version 1.8.0; scikit-learn developers, Paris, France), which internally generates out-of-fold predictions from the base learners during training to fit the meta-learner, thereby reducing the risk of overfitting.

Hyperparameter optimization via GridSearchCV was applied exclusively to XGBoost within the nested cross-validation procedure, as it was designated as the primary model for calibration and interpretability analysis. The remaining models were evaluated using default or commonly reported hyperparameter settings and served as baseline comparisons rather than fully optimized alternatives. This represents a limitation of the comparative analysis, as fully tuned versions of all models may yield different relative performance.

#### 3.2.1. Preprocessing Pipeline

The implementation of all preprocessing activities through a single machine learning pipeline system which uses the scikit-learn Pipeline and ColumnTransformer components was necessary to maintain research methods and safeguard against data breaches.

The system automatically classified predictor variables into numerical and categorical feature groups according to their respective data types. The system standardized numerical variables through z-score normalization using StandardScaler, while it transformed categorical variables through one-hot encoding which ignored unknown categories that appeared during cross-validation.

The pipeline processing embedded preprocessing functions to handle all feature transformations, learning from training folds during cross-validation. The system design protects against data leakage while evaluating models according to their actual performance on data that was not used for training.

The system developed an end-to-end modeling framework which integrated the preprocessing pipeline with the classifier system. The system maintained complete boundary separation between training and validation processes because model evaluation and hyperparameter tuning used the entire pipeline, which included all elements of the system.

#### 3.2.2. Handling Class Imbalance

The dataset showed moderate class imbalance because only 11.16% of encounters led to 30-day readmission. The imbalanced clinical dataset can bias predictive models toward the majority class while underestimating minority-class risk.

The dataset exhibited moderate class imbalance (11% positive cases); therefore, evaluation metrics beyond ROC–AUC, such as precision–recall AUC (PR–AUC), were also reported to provide a more informative assessment of model performance under class imbalance.

The researchers used cost-sensitive learning through XGBoost to solve class imbalance problems instead of using synthetic resampling methods. The researchers calculated scale_pos_weight by determining the negative and positive class frequency ratio which they used as a training parameter for the classifier. The approach raises the penalty which applies to misclassified minority-class observations (readmissions) while maintaining the original data distribution intact.

To ensure a consistent and fair comparison across all models, class-weighted strategies were also applied to all baseline classifiers. Logistic Regression, Random Forest, and LightGBM were configured with class_weight = “balanced”, which automatically adjusts class weights inversely proportional to their frequencies in the training data. Both scale_pos_weight and class_weight = “balanced” achieve the same objective of penalizing misclassification of the minority class, and represent the standard class imbalance handling mechanisms for their respective classifiers within the scikit-learn framework.

This approach was deliberately chosen over resampling-based alternatives such as SMOTE, ADASYN, or ENN for several reasons. First, resampling methods generate synthetic samples based on feature-space interpolation, which may produce clinically implausible patient profiles in datasets with mixed feature types such as ours. Second, cost-sensitive learning preserves the natural class prevalence of 11%, which reflects real-world readmission incidence and is essential for accurate probability calibration. Third, embedding scale_pos_weight directly within the classifier inside the pipeline guarantees zero data leakage across all cross-validation folds, unlike resampling methods applied outside the cross-validation loop.

It is important to distinguish between bias correction and robustness assessment. While nested cross-validation and bootstrap resampling assess performance consistency and statistical uncertainty, they do not correct for class imbalance bias. In this study, bias correction was achieved through cost-sensitive learning via the scale_pos_weight parameter, which directly adjusts the loss function during training to penalize misclassification of the minority class. This approach addresses the source of bias at the model level without requiring data-level resampling prior to training.

#### 3.2.3. Nested Cross-Validation

The researchers used nested cross-validation as their primary evaluation method because it provides an impartial assessment of model performance while reducing the chances of seeing false positive results. Nested cross-validation establishes itself as the most reliable evaluation method used in predictive modeling because it protects against data leakage between model selection procedures and performance evaluation processes, which occur during the process of hyperparameter optimization.

The outer loop consisted of a stratified 5-fold cross-validation procedure. The dataset was divided into two parts during each outer fold with an 80% training subset and a 20% independent testing subset, while maintaining the original class distribution of the data. The outer fold which the team withheld from testing served only as the evaluation site for performance testing because the team did not use it to optimize any hyperparameters.

The researchers conducted hyperparameter tuning through GridSearchCV by executing 3-fold stratified cross-validation within every outer training fold of their study. The hyperparameter search space included the number of trees (n_estimators), maximum tree depth (max_depth), and learning rate (learning_rate) of the XGBoost classifier. The inner loop selected models according to their area under the receiver operating characteristic curve (AUC) scores which provide an unbiased method for assessment of model performance in situations where two classes have different base rates.

The researchers completed their inner loop hyperparameter optimization process by retraining their top model on complete outer training data before testing its performance against the preserved outer test data. The procedure was executed throughout the five outer folds which produced five distinct AUC assessments.

The final nested cross-validation performance used mean AUC results from outer folds together with standard deviation measurements to assess performance stability. The modeling framework proved its strength because the folds showed low variability which matched the model performance without any overfitting.

The nested cross-validation design achieved its goal to separate performance evaluation from hyperparameter tuning which resulted in discrimination metrics that accurately measured out-of-sample performance instead of giving inflated training results.

Given the class imbalance in the dataset, the F1 score for the minority class was additionally reported as a complementary evaluation metric, defined as follows:

(1) $$Precision=\frac{TP}{\left(TP+FP\right)}\;Recall=\frac{TP}{\left(TP+FN\right)}\;F1=\frac{2\times (Precision\times Recall)}{\left(Precision+Recall\right)}$$

where TP (true positives) refers to readmitted patients correctly identified by the model, FP (false positives) refers to non-readmitted patients incorrectly flagged as high risk, and FN (false negatives) refers to readmitted patients missed by the model. This metric was computed specifically for the minority class (readmitted within 30 days) to provide a more informative assessment of model performance under class imbalance conditions.

#### 3.2.4. Final Model Training and Probability Calibration

Following nested cross-validation and selection of the optimal hyperparameter configuration, the final model used entire dataset training to achieve maximum information utilization. Discrimination metrics which include AUC measurement assess a model’s ability to rank patients according to risk but these metrics do not ensure that predicted probabilities maintain accurate calibration. Accurate probability estimation holds critical importance in clinical decision-making because treatment thresholds along with intervention strategies rely directly on predicted risk values.

Post-training calibration was conducted through Platt scaling which uses sigmoid calibration together with CalibratedClassifierCV to improve probability reliability. The method applies a logistic regression model to the classifier’s output scores which results in calibrated probabilities that better match actual outcome frequencies.

The Brier score served as the evaluation method for calibration performance because it calculates the average squared difference between predicted probabilities and real outcomes. The accuracy of probability predictions improves when Brier scores decrease. The calibrated AUC results confirmed that the system maintained its ability to distinguish between different classes after the probability values were modified.

The final model produces two outcomes through its use of probability calibration which allows the system to both accurately rank patients according to their risk levels and produce probability estimates that healthcare providers can use to make decisions based on specific thresholds.

#### 3.2.5. Bootstrap Resampling Procedure

Nonparametric bootstrap resampling enabled researchers to measure statistical uncertainty while testing how well their models performed. Bootstrap analysis delivers more complete performance assessment through its multiple metrics because it shows testing results under various sampling conditions which create different measurement outcomes.

Researchers created 1000 bootstrap samples by extracting data from the complete dataset through random selection. The team used the calibrated model predictions to assess discrimination and calibration performance with every resampled dataset. The team conducted three measurements for each bootstrap iteration which included AUC and Brier score and RR between specific risk groups.

The 95% confidence intervals (CI) for each metric were derived from the 2.5th and 97.5th percentiles of the bootstrap distributions. The method utilizes percentiles to establish performance metrics because it does not require distributional assumptions which suit complex machine learning models.

Bootstrap-derived confidence intervals show how stable and reproducible the model performs its predictions. The resampled cohorts produced narrow intervals which demonstrated consistent performance, while wide intervals showed that the model might react differently to different sampling methods. The statistical credibility of the findings increases when confidence intervals get reported with point estimates, which also follows established standards for predictive modeling research.

#### 3.2.6. Clinical Utility Evaluation

While discrimination and calibration metrics quantify statistical performance, they do not directly indicate whether a model provides clinical benefit. To evaluate practical decision-making value, clinical utility was assessed using Decision Curve Analysis (DCA) and Clinical Impact Curve (CIC) methodologies.

##### Decision Curve Analysis

Decision Curve Analysis was performed to evaluate the net clinical benefit of the model across a range of threshold probabilities [23]. Net benefit incorporates both true positive and false positive classifications while accounting for the relative harm of unnecessary interventions.

For a given threshold probability $p_{t}$, patients with predicted risk ≥ $p_{t}$ were considered high risk. Net benefit was calculated as:

(2) $$Net\;Benefit=\frac{TP}{N}-\frac{FP}{N}\times \frac{p_{t}}{1-p_{t}}$$

where *TP* represents true positives, *FP* represents false positives, and *N* is the total sample size.

The model’s net benefit was compared against two reference strategies:

- Treat-All (intervening on all patients),
- Treat-None (no intervention).

Decision curves were generated across threshold probabilities ranging from 1% to 30%, reflecting clinically plausible intervention thresholds for hospital readmission prevention programs.

A model was considered clinically useful when its net benefit exceeded both reference strategies across a relevant threshold range. This analysis determines whether using the model improves decision-making beyond naive treatment policies.

##### Clinical Impact Curve

To further quantify real-world implications, a Clinical Impact Curve was generated. This analysis estimates, per 1000 patients:

- The number of individuals classified as high risk at each threshold,
- The number of true readmissions among those classified high risk.

This representation provides a direct interpretation of model consequences at scale, enabling healthcare administrators to understand how many patients would be flagged for intervention and how many of those would truly experience readmission.

##### Threshold-Based Clinical Metrics

In addition to threshold-independent measures (AUC), threshold-dependent clinical metrics were calculated at a predefined 10% risk threshold, chosen to approximate the observed prevalence and represent a realistic intervention cutoff.

At this threshold, the following metrics were computed:

- Sensitivity (recall)
- Specificity
- Positive Predictive Value (PPV)
- Negative Predictive Value (NPV)
- Number of high-risk patients identified per 1000
- Number of true readmissions per 1000

These measures provide interpretable performance indicators aligned with operational decision-making in hospital readmission reduction programs.

By combining discrimination, calibration, and clinical utility analyses, the evaluation framework extends beyond predictive accuracy to assess whether the model meaningfully improves patient stratification and healthcare resource allocation.

#### 3.2.7. Model Explainability and Stability Analysis

To enhance transparency and facilitate clinical interpretability, model explainability was assessed using SHapley Additive exPlanations (SHAP). SHAP is a game-theoretic approach that decomposes model predictions into additive feature contributions, allowing both global and local interpretation of complex machine learning models [24].

Given the nonlinear and ensemble-based structure of XGBoost, SHAP values were computed using the TreeExplainer algorithm, which is specifically optimized for tree-based models. SHAP values quantify the marginal contribution of each feature to the predicted probability of 30-day readmission.

##### Global Feature Importance

Global interpretability was assessed by aggregating absolute SHAP values across all observations to determine the most influential predictors. This analysis identifies features that consistently drive risk prediction at the population level.

Summary plots were generated to visualize:

- The relative magnitude of feature importance,
- The directionality of effect (whether higher feature values increase or decrease predicted readmission risk),
- The distribution of feature contributions across the cohort.

This approach ensures that model behavior aligns with known clinical risk factors and does not rely on spurious associations.

##### Stability Analysis of SHAP Rankings

To assess the robustness of feature importance rankings, a stability analysis was conducted using bootstrap resampling.

The model was retrained on 100 bootstrap samples of the dataset, and for each iteration, the top 10 features based on mean absolute SHAP value were recorded. Feature stability was quantified as the percentage of bootstrap iterations in which a given feature appeared among the top-ranked predictors.

Stable predictors—those consistently appearing across resampled datasets—indicate that model explanations are not driven by sampling noise or random fluctuations. Conversely, unstable features may reflect sensitivity to cohort composition and reduced interpretive reliability.

This stability analysis strengthens confidence in the model’s interpretability and ensures that reported clinical drivers of readmission risk are reproducible rather than artifacts of a single training instance.

By integrating SHAP-based explainability with bootstrap stability assessment, the study ensures that predictive performance is complemented by transparent and reproducible interpretation—an essential requirement for translation into clinical decision-support systems.

## 4. Results

This section presents the performance evaluation of the proposed machine learning approach for predicting 30-day hospital readmission among patients with diabetes. Model performance was assessed using multiple complementary metrics that evaluate discrimination, calibration, and clinical utility. The evaluation framework included nested cross-validation to estimate generalization performance, probability calibration to improve risk estimation accuracy, and bootstrap resampling to assess statistical robustness.

Table 3 presents the discriminative performance of several baseline machine learning models, all evaluated under the same nested cross-validation protocol (5 outer folds, 3 inner folds) and preprocessing pipeline used for XGBoost, ensuring a consistent and fair comparison framework. Logistic Regression achieved an ROC–AUC of 0.657, while Random Forest obtained an ROC–AUC of 0.650. The XGBoost model achieved an ROC–AUC of 0.664, and LightGBM achieved an ROC–AUC of 0.660. The stacking ensemble achieved the highest ROC–AUC of 0.665, slightly outperforming the individual models. However, XGBoost was retained as the primary model for interpretability and calibration analysis. Based on this comparison, Table 4 summarizes the detailed performance metrics of the selected XGBoost model.

Table 4 summarizes the overall performance of the proposed prediction model using multiple evaluation metrics. The results indicate that the model achieved acceptable discriminative ability and good calibration performance.

Because the dataset exhibited moderate class imbalance (11% positive cases), precision–recall AUC (PR–AUC) was also reported as a complementary metric to ROC–AUC, providing a more informative assessment of minority-class prediction performance.

Precision–recall analysis yielded a PR-AUC of 0.215, indicating improved performance compared with the baseline prevalence of 11%.

### 4.1. Discriminative Performance: Nested Cross-Validation

The nested cross-validation method first assessed model discrimination to provide an unbiased estimate of generalization performance. The XGBoost model showed consistent results across the five outer folds, achieving a mean area under the receiver operating characteristic curve (AUC) of 0.664 with low standard deviation between folds.

The low variability across outer folds indicates that the model maintained stable performance and did not suffer from major overfitting. Because hyperparameter tuning was performed exclusively within the inner cross-validation loop, the reported estimates reflect true out-of-sample discrimination rather than optimistic training performance.

The AUC demonstrates moderate discriminative ability, which is consistent with the inherent complexity and heterogeneity of hospital readmission data. Readmission prediction in large administrative datasets depends on multiple clinical and social determinants, which generally results in lower discrimination compared with highly controlled diagnostic datasets.

This performance is consistent with previously published models using the same dataset, where reported AUC values range from 0.661 to 0.667, suggesting that the proposed framework achieves competitive discrimination despite employing more rigorous nested cross-validation methodology

Given the class imbalance, the F1 score for the minority class was additionally reported. The XGBoost model achieved an F1 score of 0.27 for the readmitted class, reflecting the inherent difficulty of identifying true readmissions in a heavily imbalanced dataset where positive cases represent only 11.16% of encounters.

### 4.2. Final Model Performance and Calibration

After completing nested validation, the final model was retrained on the full dataset, and Platt scaling was applied to calibrate its probability estimates.

Following calibration, the model achieved an AUC of approximately 0.688, indicating that discriminative performance was preserved. The Brier score was approximately 0.094, demonstrating good overall probability accuracy.

The calibration process improved the alignment between predicted readmission probabilities and observed outcome rates, thereby enhancing the reliability of risk estimation. This adjustment increases clinical usability, as it enables decision-making based on predefined absolute risk thresholds.

It should be noted that the nested cross-validation AUC (0.664) and the full-dataset AUC (0.688) are not directly comparable, as they are derived using different evaluation strategies. The nested CV AUC reflects out-of-sample performance across five independent folds, while the full-dataset AUC is computed after retraining the final model on the complete dataset. The observed difference therefore reflects the additional information available when training on the full dataset rather than an effect of probability calibration itself, which by definition does not alter the rank order of predictions or the AUC.

### 4.3. Bootstrap Confidence Intervals

Statistical robustness was evaluated using 1000 bootstrap resamples. The 95% confidence interval for AUC was narrow, indicating consistent discriminative performance across resampled datasets. Similarly, the Brier score confidence interval showed limited variability, supporting the reliability of predicted probabilities.

The relative risk (RR) between high-risk and low-risk groups ranged from approximately 3.9 to 4.0, with a narrow 95% confidence interval. The bootstrap-derived intervals demonstrate that model performance remained stable and reproducible across the studied population.

### 4.4. Risk Stratification

Patients were stratified into tertiles based on predicted risk (low, medium, high).

Observed 30-day readmission rates were:

- Low-risk group: approximately 4–5%;
- Medium-risk group: approximately 9–10%;
- High-risk group: approximately 18–19%.

This clear monotonic gradient demonstrates effective risk separation. Patients in the high-risk group had nearly four times the risk of readmission compared to those in the low-risk group.

Such stratification may support targeted intervention strategies by identifying subpopulations with substantially elevated risk.

### 4.5. Decision Curve Analysis

**Figure 1:**
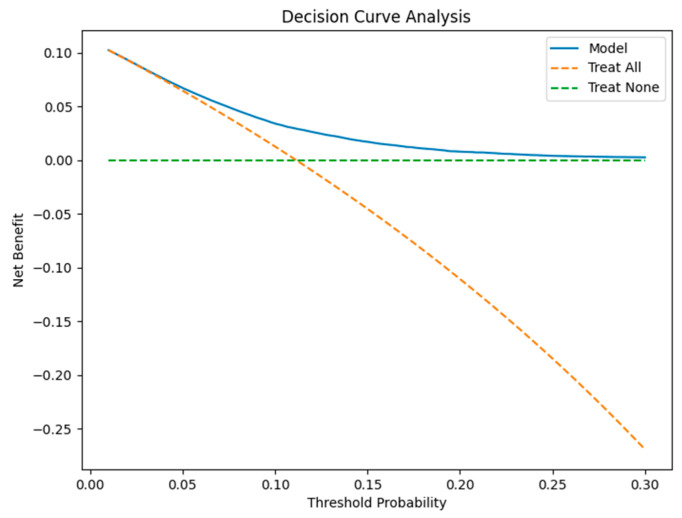
Decision curve analysis showing net benefit of the model compared with Treat-All and Treat-None strategies across threshold probabilities from 1% to 30%.

Decision curve analysis confirmed that the model provided net benefit across clinically relevant threshold probabilities ranging from approximately 1.6% to 30%. As illustrated in Figure 1, the model consistently outperformed both the “Treat-All” and “Treat-None” strategies across this threshold range.

The net benefit curve of the model remained above the reference strategies, indicating that model-guided intervention would yield better outcomes than non-stratified approaches. These findings demonstrate that the model’s value extends beyond discrimination and translates into meaningful clinical utility.

### 4.6. Threshold-Based Performance Analysis

To demonstrate the inherent trade-off between sensitivity and positive predictive value across clinically plausible decision thresholds, model performance was evaluated at multiple operating points ranging from 5% to 30% risk. Table 5 summarizes the key metrics at each threshold.

As shown, lower thresholds yield higher sensitivity but substantially lower PPV, resulting in a greater number of patients flagged for intervention. Higher thresholds improve PPV but at the cost of missing a larger proportion of true readmissions. Clinicians and administrators should select the operating threshold based on the relative costs of false positives and false negatives in their specific institutional context.

### 4.7. Clinical Impact Curve

**Figure 2:**
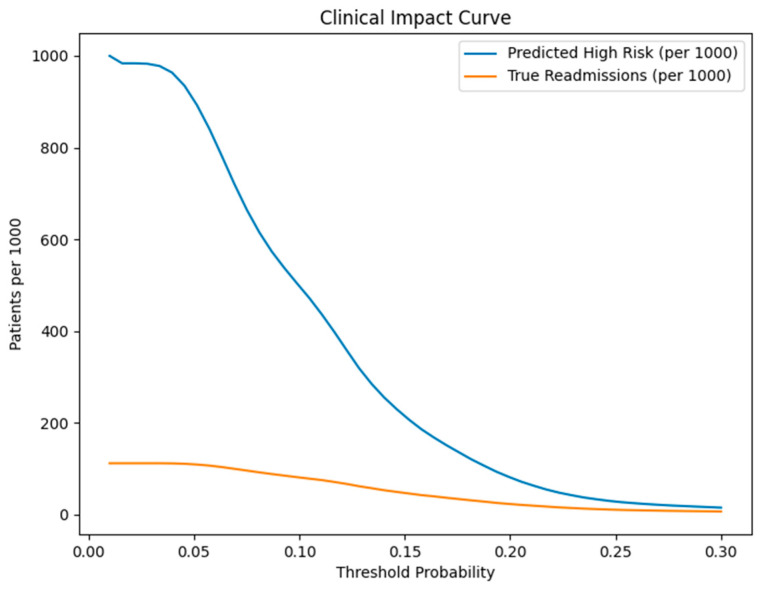
Clinical impact curve illustrating the number of patients classified as high risk per 1000 and the corresponding number of true readmissions across threshold probabilities.

The clinical impact curve quantified real-world implications per 1000 patients. As shown in Figure 2, the number of patients classified as high risk decreases progressively as the decision threshold increases, while the number of true readmissions among those classified remains proportionally lower.

At a 10% risk threshold:

- Approximately 500 patients per 1000 were classified as high risk,
- Approximately 80 true readmissions per 1000 were correctly identified.

At this threshold, sensitivity was approximately 72%, and negative predictive value exceeded 90%, indicating that the model effectively identifies the majority of readmissions while maintaining strong reassurance for low-risk patients. Although the positive predictive value remains modest—consistent with the low prevalence of readmission—the model substantially improves enrichment compared to baseline prevalence.

### 4.8. Model Explainability and Stability

**Figure 3:**
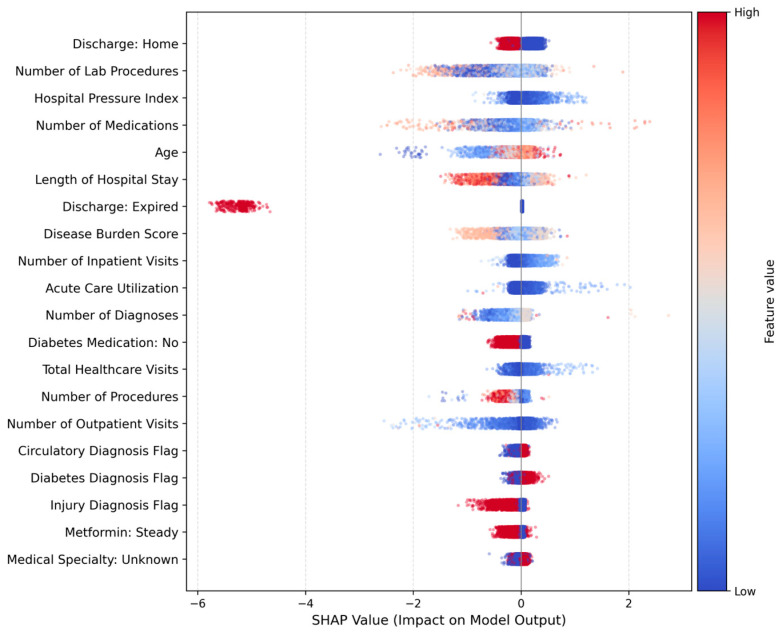
SHAP summary plot displaying global feature importance and directionality of effect for the final XGBoost model.

SHAP analysis (version 0.50.0; Lundberg & Lee, open source) identified clinically plausible predictors as major drivers of readmission risk. As presented in Figure 3, the summary plot highlights the relative importance and directionality of key predictors within the final XGBoost model. Number of prior encounters,

- Number of diagnoses,
- Length of hospital stay,
- Age,
- Number of medications.

The directionality of SHAP contributions was consistent with known clinical patterns, with higher comorbidity burden and greater healthcare utilization associated with increased readmission probability.

Stability analysis demonstrated that the most influential predictors appeared consistently across bootstrap iterations, with several features appearing in 100% of resampled top-10 rankings. This finding indicates that model explanations are robust and not driven by sampling noise.

### 4.9. Precision–Recall Performance

**Figure 4:**
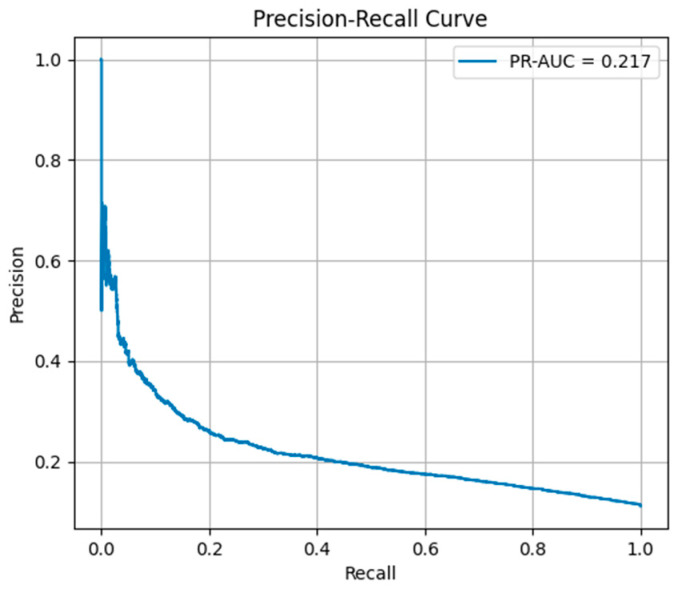
Precision–Recall Curve.

Because the dataset is highly imbalanced (11% positive class), precision–recall analysis was also conducted. The model achieved a PR-AUC of 0.215, which substantially exceeds the baseline prevalence of 0.111, as illustrated in Figure 4.

This indicates that the model effectively enriches the identification of high-risk patients compared with random classification.

### 4.10. Clinical Utility of the Prediction Model

The decision curve analysis demonstrated that the proposed model provides a positive net clinical benefit across a wide range of threshold probabilities. This suggests that the model could be used as a decision-support tool to identify high-risk patients and prioritize preventive interventions.

## 5. Discussion

The study developed a machine learning approach which aims to predict 30-day hospital readmission rates for diabetic patients by using data from multiple medical institutions. The proposed method uses nested cross-validation and probability calibration together with bootstrap confidence intervals and clinical utility analysis and SHAP-based interpretability to create a new evaluation system which evaluates historical accuracy but now contains actual clinical value.

The model showed its ability to discriminate between groups through a performance rating of moderate strength which achieved an AUC of approximately 0.664 through nested cross-validation and an AUC of approximately 0.688 through calibration testing. This level of discrimination shows a better outcome than single-center datasets which contain highly curated data, but matches the established difficulty of predicting hospital readmission rates. The factors which determine readmission include comorbidity burden and healthcare utilization patterns and discharge planning quality and unmeasured social factors. The unpredictability of outcomes appears in moderate AUC values which show up in large administrative datasets because these datasets only partially include clinical details and social determinants.

The stability demonstrated through nested cross-validation and bootstrap resampling tests shows that the model achieves consistent results which do not depend on changes in sample data. The research findings achieve stronger evidence through the narrow confidence intervals which measure discrimination and relative risk. The research achieves stable results because clinical prediction research needs to develop accurate assessments of patient outcomes while preventing direct overfitting and optimistic result reporting.

The calibration analysis showed that hospitals improved their readmission rate predictions because their predicted probabilities better matched actual readmission rates. The calibrated Brier score shows that probability estimation achieves reliable results, which hospitals need to make decisions based on treatment thresholds. The hospital system uses predefined risk thresholds to activate patient interventions, which makes accurate probability estimation essential for practical implementation.

The model demonstrates positive clinical utility through its net benefits which extend across multiple clinically important threshold evaluations. The decision curve analysis proved more effective than both the “Treat-All” and “Treat-None” approaches because model-based treatment solutions enhanced both patient assignment and medical resource distribution. The clinical impact curve demonstrated that risk levels lead to specific operational results, which resulted in 1000 patient interactions showing both high-risk patient detection and actual readmission occurrences.

The risk stratification analysis revealed a consistent pattern, which showed that high-risk individuals had four times greater readmission rates than low-risk individuals. The two groups should use this method to support their particular intervention strategies, which demonstrate how this model can help reduce readmissions.

The SHAP-based explainability showed that the model used clinically valid predictions which included previous medical treatment records and existing medical conditions and hospitalization duration. The SHAP ranking stability analysis showed that essential model predictors appeared consistently throughout all bootstrap testing which proved that the model explanations remained stable instead of showing random variations. The clinical implementation requires this interpretability because doctors trust transparent models which assist their work with decision-support systems.

Although the discriminative performance of the proposed model is comparable to conventional index-based methods, the value of the machine learning approach lies beyond AUC. Unlike fixed scoring systems, the proposed approach provides calibrated probability estimates, decision curve analysis demonstrating net clinical benefit, and SHAP-based interpretability that identifies patient-specific risk drivers. These capabilities support more individualized clinical decision-making than is possible with index-based tools.

### 5.1. Comparison with Previous Studies

Previous studies on hospital readmission prediction have frequently reported AUC values ranging between 0.60 and 0.75 when using administrative or structured electronic health record data. Higher performance metrics are often observed in single-center datasets or highly curated cohorts; however, such results may not generalize well to heterogeneous, multi-institutional populations.

In contrast, the present study employed a large, multi-hospital dataset comprising over 100,000 encounters, reflecting real-world clinical variability. The moderate but stable discrimination observed in this study aligns with prior large-scale readmission models and reinforces the notion that readmission is inherently difficult to predict using structured data alone. Notably, studies using this exact dataset—including Emi-Johnson et al. [15] who reported an XGBoost AUC of 0.667, and Shang et al. [18] who reported an AUC of 0.661—demonstrate that our calibrated AUC of 0.688 represents the upper bound of what has been achieved on this data source. Importantly, the methodological rigor applied here—particularly nested cross-validation, bootstrap confidence intervals, and probability calibration—reduces the risk of optimistic bias that may inflate reported performance in less robust studies.

It is important to acknowledge that the studies included in Table 5 are not directly comparable due to fundamental differences in population size, cohort composition, and evaluation metrics. For instance, Mishra et al. reported an AUC of 0.94 on a highly homogeneous cohort of only 352 patients—a scenario well-known to produce inflated and non-generalizable performance estimates. Similarly, Silva et al. focused on pediatric patients, and Awe et al. reported R^2^ rather than AUC, making direct performance comparisons inappropriate for a binary classification task. These studies are included for contextual purposes only, not as performance benchmarks. Furthermore, the available evidence suggests that the performance ceiling for readmission prediction using administrative data alone is approximately AUC 0.70. Meaningful improvements beyond this ceiling are unlikely to come from more complex models applied to the same limited features, but rather from incorporating richer data sources such as social determinants of health, laboratory values, and post-discharge information.

Unlike many prior investigations that focus primarily on discrimination metrics, this study incorporated calibration assessment, decision curve analysis, and clinical impact evaluation. These additional layers of validation provide a more comprehensive assessment of clinical utility rather than relying solely on AUC as a performance indicator.

The results in Table 6 demonstrate that the proposed model achieves predictive performance that matches the established results which show how hospital readmissions can be predicted through structured electronic health record data. The previous research studies demonstrated high performance results which researchers obtained from testing smaller or more homogeneous study groups because these limitations affected their ability to apply results to larger healthcare populations. The current research employed a dataset which included more than 100,000 patient encounters from 130 different hospitals to study various medical conditions.

The achieved discrimination results with AUC values between 0.664 and 0.688 demonstrate moderate performance which matches results from other large-scale research studies using this dataset and similar administrative data sources. The current research expands previous studies by using a more comprehensive evaluation method which includes nested cross-validation together with probability calibration and bootstrap confidence intervals and decision curve analysis for clinical utility assessment. The methodological elements enable researchers to conduct model performance assessment together with clinical applicability evaluation through more reliable testing methods which extend beyond standard discrimination metric evaluations.

These findings highlight that while predictive performance remains comparable to previous studies, the proposed approach emphasizes a more comprehensive evaluation pipeline, incorporating nested cross-validation, calibration, and decision curve analysis, rather than claiming superior predictive performance over prior studies, which differ in population, dataset, and evaluation strategy.

### 5.2. Strengths of the Study

This research study develops particular advantages that differentiate it from typical applications of machine learning in healthcare settings.

The nested cross-validation method establishes a complete separation between hyperparameter tuning and performance estimation which prevents any optimistic evaluation results. This approach establishes higher confidence in the discrimination metrics that the study reports.

The study established probability calibration as an essential method because clinical models need accurate risk assessments which go beyond simple ranking methods.

The combination of bootstrap confidence intervals with the research study builds statistical power which demonstrates how the model produces consistent results and maintains its ability to assess relative risk differences.

The study used decision curve analysis and clinical impact curves to assess clinical utility which showed how statistical results would affect actual medical practice. Healthcare implementation requires this process, which machine learning studies tend to skip.

The combination of SHAP-based model explanations with stability analysis produces model descriptions that medical professionals can trust while showing how the system works to potential users of clinical decision-support systems.

### 5.3. Clinical Implications

The model shows potential value in clinical settings because it helps hospitals decrease their patient readmission rates. The risk levels studied show a consistent pattern which indicates that high-risk patients should receive more comprehensive discharge planning together with post-discharge monitoring and medication reconciliation and case management support.

The model improves identification of high-risk patients because its positive predictive value shows low rates of readmission which makes the high-risk group more accurate than the original baseline. The high negative predictive value indicates that healthcare providers can reduce treatment needs for low-risk patients which helps them use their resources more effectively.

The decision curve analysis shows that the clinical utility of this system demonstrates that model-based decision-making creates more overall advantages than standard methods across different levels of treatment assessment.

### 5.4. Limitations

Several limitations should be acknowledged.

Data characteristics: The dataset covers the period 1999–2008, predating the Hospital Readmission Reduction Program (HRRP) introduced in 2012, which fundamentally changed hospital incentives and readmission patterns. The model should therefore be considered a historical artifact rather than a directly deployable contemporary tool. Additionally, the dataset does not differentiate between planned and unplanned readmissions, which may introduce noise into the outcome definition.

Missing clinical information: The reliance on administrative data represents a meaningful limitation. Key determinants of readmission—including laboratory values, medication adherence, social determinants of health, and post-discharge care—are not captured. Critically, these are not merely missing variables; they are likely the primary drivers of readmission risk, meaning the model fundamentally predicts a surrogate (prior utilization) rather than true underlying risk. This distinction has important implications for clinical interpretation.

Methodological limitations: The cross-validation strategy did not enforce patient-level splits, meaning encounters from the same patient may appear across training and test folds. Future work should consider GroupKFold strategies. Additionally, decision curve analysis was computed using full-dataset predictions rather than strictly out-of-sample estimates. The creation of binary diagnostic flags collapses ordinal information about primary versus secondary diagnoses; future work should explore weighted diagnostic features.

Algorithmic bias: No subgroup analysis was conducted to evaluate model performance across age groups, genders, or racial/ethnic categories. Such analyses are a critical requirement for any model intended for clinical use, as differential performance across subgroups could exacerbate existing healthcare disparities.

Generalizability: Internal robustness was demonstrated through nested cross-validation and bootstrap resampling; however, external validation across modern multi-center cohorts is required before clinical deployment.

### 5.5. Future Directions

The upcoming studies will utilize new research data which will include time-based laboratory results and medication adherence data and socioeconomic information to improve prediction results. The assessment of operational effects will require both real-time electronic health record system integration and ongoing validation research.

The use of federated learning as a privacy-preserving technology will enable multiple institutions to work together without needing to share patient data, which will help create better research results while keeping patient information secure.

## 6. Conclusions

Predicting 30-day hospital readmission in diabetic patients is inherently difficult. Readmission risk reflects a combination of clinical history, healthcare utilization patterns, and social circumstances that administrative data can only partially capture. This study did not set out to overcome those constraints, but to work within them more carefully than is common in the literature.

The model achieved a calibrated AUC of 0.688 and a Brier score of 0.094, consistent with prior work on this dataset. These estimates were produced under a nested cross-validation design that separated hyperparameter tuning from performance evaluation, reducing the optimistic bias that affects many published readmission models. Probability calibration, decision curve analysis, and bootstrap confidence intervals were treated as essential components of evaluation rather than optional additions.

Risk stratification showed meaningful separation between patient groups, with high-risk patients readmitted at nearly four times the rate of low-risk patients. SHAP analysis identified prior utilization, comorbidity burden, and length of stay as the dominant predictors—findings consistent with clinical expectations and supportive of the model’s interpretability.

However, there are real limitations. The data predates the Hospital Readmission Reduction Program, planned and unplanned readmissions cannot be distinguished, social determinants are absent, and no subgroup analysis was conducted. These are not peripheral concerns; instead, they define the boundaries of what this model can and cannot tell us.

What this work offers is a replicable evaluation template, not a clinical tool. External validation on contemporary data remains the necessary condition for any consideration of real-world deployment.

## Figures and Tables

**Table 1:** Missing Data Audit: Extent of Missingness and Handling Strategy for All Affected Variables.

Variable	Missing (%)	Action Taken
weight	96.86%	Removed
max_glu_serum	94.75%	Retained—imputed as “Unknown”
A1Cresult	83.28%	Retained—imputed as “Unknown”
medical_specialty	49.08%	Retained—imputed as “Unknown”
payer_code	39.56%	Removed
race	2.23%	Retained—imputed as “Unknown”
diag_3	1.40%	Retained—imputed as “Unknown”
diag_2	0.35%	Retained—imputed as “Unknown”
diag_1	0.02%	Retained—imputed as “Unknown”

**Table 2:** Dataset descriptions used in the proposed system.

Variable	Description
Dataset Name	Diabetes 130-US Hospitals (1999–2008)
Source	UCI Machine Learning Repository
Number of Hospitals	130
Total Encounters	101,766
Study Population	Hospitalized patients diagnosed with diabetes mellitus
Prediction Task	30-day hospital readmission
Positive Class (Readmitted < 30 days)	11,357 (11.16%)
Negative Class	90,409 (88.84%)
Class Imbalance Ratio	Approximately 1:8
Final Number of Predictors	55

**Table 3:** Performance comparison of baseline machine learning models.

Model	ROC-AUC
Logistic Regression	0.657
Random Forest	0.650
XGBoost	0.664
LightGBM	0.660
Stacking	0.665

**Table 4:** Summary of model performance metrics. Metrics correspond to the calibrated XGBoost model.

Metric	Value
Nested Cross-Validation ROC–AUC	0.664
Full-dataset AUC (post-calibration retraining)	0.688
Brier Score	0.094
PR–AUC	0.215
F1 Score (minority class)	0.27
Sensitivity (10% threshold)	0.723
Specificity (10% threshold)	0.530
PPV (10% threshold)	0.162
NPV (10% threshold)	0.938
Predicted High Risk per 1000	497.9
True Readmissions per 1000	80.6

**Table 5:** Model Performance Across Decision Thresholds.

Threshold	Sensitivity	Specificity	PPV	NPV	High Risk per 1000
0.05	1.000	0.073	0.119	1.000	934.8
0.10	0.881	0.524	0.189	0.972	521.0
0.15	0.570	0.839	0.308	0.940	206.5
0.20	0.264	0.967	0.501	0.913	58.9
0.25	0.108	0.994	0.707	0.899	17.1
0.30	0.038	0.999	0.886	0.892	4.8

**Table 6:** Contextual summary of related studies on hospital readmission prediction in diabetes.

Study	Dataset/Population	Methods	Evaluation Strategy	Reported Performance
Mishra et al.	352 patients from a diabetes specialty clinic	Logistic Regression, Decision Tree, Random Forest, XGBoost	Train–test split	RF AUC = 0.94; Precision = 0.84; Recall = 0.87; F1 = 0.85
Silva et al.	9080 pediatric hospitalized patients	Logistic Regression, Decision Tree, Random Forest, Gradient Boosting, XGBoost	Train/test with cross-validation	AUC = 0.814; Sensitivity = 0.81; Specificity = 0.66
Awe et al.	Massachusetts statewide hospital dataset	Ridge Regression, Random Forest, Gradient Boosting	Standard train/test evaluation	Gradient Boosting R^2^ ≈ 0.81
Emi-Johnson et al.	UCI Diabetes 130-US Hospitals dataset (101,766 records)	Logistic Regression, Random Forest, XGBoost, Deep Neural Network	Train/test evaluation	XGBoost AUC = 0.667
Liu et al.	UCI Diabetes dataset (101,766 encounters)	RF, XGBoost, SVM, KNN, Naïve Bayes, AdaBoost, MLP, LSTM	Train/test split with SMOTE	RF Accuracy = 0.88; F1 = 0.83
Shang et al.	Health Facts diabetes dataset (>100,000 records)	Random Forest, Naïve Bayes, Tree Ensemble	80/20 train/test split	AUC ≈ 0.661
Li et al.	MIMIC-III database (Medicare patients)	LSTM deep learning	Train/test evaluation	AUC ≈ 0.70
**Proposed Study**	UCI Diabetes dataset (101,766 encounters; 130 hospitals)	Logistic Regression, RF, XGBoost, LightGBM, Stacking	**Nested cross-validation + calibration + bootstrap + DCA**	**AUC = 0.664 (nested CV), calibrated AUC = 0.688; Brier = 0.094**

## Data Availability

The datasets used in this study are publicly available and can be accessed via the Kaggle platform (Google LLC, San Francisco, CA, USA) at https://www.kaggle.com/datasets/ashishkumarjayswal/diabetes-dataset (accessed on 21 August 2025) and the UCI Machine Learning Repository at https://archive.ics.uci.edu/dataset/296/diabetes+130-us+hospitals+for+years+1999-2008 (accessed on 21 August 2025). No new data were generated during this study. All analyses were conducted using these openly available datasets in compliance with the original data usage terms.

## Supplementary Materials

The following supporting information can be downloaded at: https://www.mdpi.com/article/10.3390/healthcare14091185/s1, Table S1: Feature Engineering Summary: Variable Transformations Across Preprocessing Stages.
