# Supplementary material for: A Machine Learning Approach for Predicting 30-Day Hospital Readmission in Patients with Diabetes
Source: Healthcare (Basel). 2026 Apr 28;14(9):1185. doi: 10.3390/healthcare14091185 (PMC13163415; doi:10.3390/healthcare14091185)
Supplement: Supplementary file 1 [file healthcare-14-01185-s001.zip › healthcare-4216950-supplementary.pdf]

| Table S1. Feature Engineering Summary: Variable Transformations Across Preprocessing Stages | | | |
|---|---|---|---|
| **Variable Name** | **Stage** | **Action** | **Notes** |
| **ORIGINAL DATASET (UCI — 50 variables)** | | | |
| encounter_id | Cleaning | Removed | Unique identifier — removed to prevent data leakage |
| patient_nbr | Cleaning | Removed | Patient identifier — removed to prevent data leakage |
| readmitted | Cleaning | Removed / Recoded | Recoded into binary target variable (1 = readmitted <30 days, 0 = otherwise) |
| weight | Cleaning | Removed | ~97% missing values |
| payer_code | Cleaning | Removed | High missingness |
| **AFTER CLEANING (46 variables)** | | | |
| diag_1 | Feature Engineering | Removed / Grouped | ICD codes grouped into clinical categories; replaced by binary flags |
| diag_2 | Feature Engineering | Removed / Grouped | ICD codes grouped into clinical categories; replaced by binary flags |
| diag_3 | Feature Engineering | Removed / Grouped | ICD codes grouped into clinical categories; replaced by binary flags |
| admission_type_id | Feature Engineering | Removed / Grouped | Grouped into: Emergency, Urgent, Elective, Other |
| discharge_disposition_id | Feature Engineering | Removed / Grouped | Grouped into: Home, Transfer, Expired, Other |
| admission_source_id | Feature Engineering | Removed / Grouped | Grouped into: Emergency Room, Physician Referral, Transfer, Other |

| VARIABLES ADDED BY FEATURE ENGINEERING | | | |
|---|---|---|---|
| has_diabetes | Feature Engineering | Added | Binary flag: diabetes diagnosis present in any of diag_1-diag_3 |
| has_circulatory | Feature Engineering | Added | Binary flag: circulatory diagnosis present in any of diag_1-diag_3 |
| has_respiratory | Feature Engineering | Added | Binary flag: respiratory diagnosis present in any of diag_1-diag_3 |
| has_digestive | Feature Engineering | Added | Binary flag: digestive diagnosis present in any of diag_1-diag_3 |
| has_injury | Feature Engineering | Added | Binary flag: injury diagnosis present in any of diag_1-diag_3 |
| admission_type | Feature Engineering | Added | Grouped categorical variable derived from admission_type_id |
| discharge_group | Feature Engineering | Added | Grouped categorical variable derived from discharge_disposition_id |
| admission_source_group | Feature Engineering | Added | Grouped categorical variable derived from admission_source_id |
| total_visits | Feature Engineering | Added | Sum of outpatient + emergency + inpatient visits |
| acute_utilization | Feature Engineering | Added | Sum of emergency + inpatient visits |
| disease_burden | Feature Engineering | Added | Sum of number_diagnoses + num_procedures |
| polypharmacy | Feature Engineering | Added | Binary flag: num_medications >= 5 |
| long_stay | Feature Engineering | Added | Binary flag: time_in_hospital >= 7 days |
| hospital_pressure | Feature Engineering | Added | Interaction term: time_in_hospital x number_inpatient |
| has_cancer | Feature Engineering | Added | cancer diagnosis present in any of diag_1–diag_3 (ICD codes 140–239) |

| | | | |
|---|---|---|---|
| **VARIABLES TRANSFORMED (retained)** | | | |
| age | Feature Engineering | Transformed | Categorical intervals converted to numeric midpoints (e.g., [60-70) = 65) |
| medical_specialty | Feature Engineering | Reduced | 73 categories reduced to top 10 + Other (11 categories total) |
| **FINAL DATASET (54 predictor variables)** | | | |

**Legend:**

| | |
|---|---|
| | Red = Variable Removed |
| | Green = Variable Added |
| | Yellow = Variable Transformed or Reduced |
| | Blue = Section Header |
